# Closely Related *Escherichia coli* Strains with Multiple Resistances Found on Co-Managed Pig Farms Despite Marked Differences in Farm Antimicrobial Drug Usage

**DOI:** 10.3390/vetsci13040309

**Published:** 2026-03-24

**Authors:** Francesca Martelli, Andrew Wales, Martina Velasova, Shaun Cawthraw, Rebecca Gosling, Luke Randall, Robert Horton, Fabrizio Lemma, Margherita Rambaldi, Fabio Ostanello, Alessia de Lucia, Roderick Card, Olivia Turner, Nathaniel Storey, Manal AbuOun, Muna Anjum

**Affiliations:** 1 Department of Bacteriology, Animal and Plant Health Agency, Addlestone, Surrey KT15 3NB, UK; shaun.cawthraw@apha.gov.uk (S.C.); robert.horton@apha.gov.uk (R.H.); fabrizio.lemma@apha.gov.uk (F.L.); roderick.card@apha.gov.uk (R.C.); manal.abuoun@apha.gov.uk (M.A.); muna.anjum@apha.gov.uk (M.A.); 2 Discipline of Comparative Biomedical Sciences, School of Veterinary Medicine, University of Surrey, Guildford, Surrey GU2 7AL, UK; a.wales@surrey.ac.uk; 3 Department of Epidemiological Sciences, Animal and Plant Health Agency, Addlestone, Surrey KT15 3NB, UK; martina.velasova@apha.gov.uk; 4 Department of Veterinary Medical Sciences, University of Bologna, Via Zamboni, 33, 40126 Bologna, Italy

**Keywords:** pig, swine, antimicrobial resistance, multi-drug resistance, biosecurity

## Abstract

Bacterial resistance on farms can be driven by antimicrobial use. Less is known about inter-farm transfer of resistance genes and their persistence under low antimicrobial pressure. Over two years and nine visits, we examined the shedding of resistant indicator bacteria (*Escherichia coli*) on one lower- and one higher-antimicrobial-usage pig farm. There was a unidirectional transfer of some less healthy pigs from the former to the latter. Faecal pools (180) were cultured on Chromagar ECC, with and without added cefotaxime or ciprofloxacin. Presumptive *E. coli* were phenotyped, and many ciprofloxacin-resistant isolates were whole-genome sequenced. Comparing farms, there was more (*p* < 0.0001) phenotypic resistance to the antimicrobial panel from the higher-usage unit, and markedly more (about ten-fold) multi-resistance. Significantly elevated individual drug resistances on this unit correlated with recently used antimicrobials. Ciprofloxacin and/or cefotaxime-resistant isolates were often present, although in low proportions. Neither of these antimicrobial classes had been administered recently, except for one fluoroquinolone course late in the study. AMR genes were more diverse from the higher-usage farm, but some resistant and multi-resistant isolates were closely related between farms. Thus, we demonstrated the maintenance of resistance genes in strains present on farms, even where selective pressure was low.

## Simple Summary

We have a limited understanding of the transfer of antibiotic-resistant bacteria between farms, and their persistence on farms when antibiotic use is minimised. Both of these phenomena may blunt attempts to reduce resistance on farms. To gain further insights into these matters, we analysed antibiotic resistance in some ‘normal’ gut bacteria (*E. coli*) from two pig farms. The first farm used much less antibiotics than the second. It also periodically transferred pigs to it. There was clear evidence that *E. coli* from the second farm showed the most frequent antibiotic resistance. However, the first farm still had a low-level reservoir of *E. coli* strains resistant to antibiotics that are deemed critically important in human medicine, despite not using such drugs. Moreover, although pigs only ever transferred from the low to the high antibiotic usage farm, DNA analysis showed that there were very closely related antibiotic-resistant *E. coli* strains on both farms. So, the amount of antibiotics used probably affected the level of resistance on the farms, but avoiding antibiotic use did not eliminate resistant bacteria. These moved between the farms, possibly with animal transfers. This evidence should inform strategies to minimise resistant bacteria in food, protecting human health and antibiotic effectiveness.

## 1. Introduction

Antimicrobial resistance (AMR) is recognised as a global threat to public health, and a One Health approach is necessary to tackle its development and spread [1]. The use of antimicrobial drugs in livestock is known to be one of the drivers for the increase in AMR not only in pathogens but also in commensal bacteria [2]. One bacterium that is often used as an indicator in monitoring AMR is *Escherichia coli*, as it is a ubiquitous faecal organism, readily cultured and characterised, and it is considered to show AMR profiles that reflect livestock population exposure to antimicrobial drugs [3,4]. Potentially, AMR profiles of commensal *E. coli* can also indicate the risk of such resistances being present in bacteria with more pathogenic capabilities within the same population [5,6]. Aggregated data within the European Union (EU) has shown a positive correlation between antimicrobial use on farms and the corresponding resistances among *E. coli* from food-producing animals for several antimicrobial (AM) classes, including quinolones and third- and fourth-generation cephalosporins [7]. These are all designated by the World Health Organisation as ‘highest priority critically important’ (HPCI) AMs [8].

A review of experimental studies in pigs concluded that increased AMR was commonly seen among *E. coli* isolates following oral administration of AMs [9]. Furthermore, several field surveys of pig units demonstrated an association between the oral administration of AMs and an increase in AMR [9,10,11,12,13,14,15]. More broadly, recent analyses of national monitoring data in the Netherlands and Belgium have documented decreases in the prevalence of resistance to certain AMs, including fluoroquinolones, among indicator *E. coli* from pigs in parallel with a declining frequency in the use of those drugs [16,17]. The method of on-farm AM administration may also affect resistance. For instance, in-feed group medication of pigs with some AMs was associated with greater resistance to that drug in *E. coli* when compared to individual oral or parenteral treatment [11]. However, the relationships between particular AMs, route(s) of administration, and associated resistances are not simple [15] and are subject to confounders such as dose, duration, and frequency of treatment, plus the number of animals treated.

Reducing or suspending the use of extended-spectrum cephalosporins in animals is associated with a reduction in the carriage of bacteria resistant to these drugs in the target livestock, and subsequently in people [18,19]. However, for some other AMs used traditionally in livestock production, the association between reduced usage and lowered prevalence of resistance is less clear [11,16,20]. Some AM resistance genes can persist in bacteria isolated from pigs on a given farm despite the use of corresponding AMs being reduced or suspended [21].

Factors such as compensatory evolution and the use of other AMs or biocides can influence such persistence [22,23]. There is also some limited published evidence of the transfer of AMR between farms, albeit often without clarity regarding routes of transfer [23]. There remains much to learn about how AMR on farms is impacted by AM usage, co-selection, and biosecurity. The recent availability of next-generation sequencing helps us investigate such matters by allowing the tracking of many strains of indicator bacteria with high discrimination, alongside analyses of genomic determinants of resistance.

The aim of the present study was to compare AMR in *E. coli* on two United Kingdom (UK) pig farms: one farm was managed to proactively reduce AM usage, whilst the other (receiving pigs suffering clinical symptoms or delayed growth) employed more frequent therapeutic use of AMs. A special circumstance was that the farms were linked by unidirectional transfers of young stock, from the low- to the high-AM-usage farm. The hypotheses were that the prevalence of AMR on the two farms would be influenced by the particular AM drugs used on each farm and how they were used (i.e., routes of administration and dosages), and that there may be temporal and/or between-premises patterns of strain-relatedness and AMR gene persistence influenced by their linkage via livestock. Longitudinal sampling and whole-genome sequencing (WGS) were key investigational techniques for the latter hypothesis.

## 2. Materials and Methods

### 2.1. Study Farms

Farm 1 (F1) comprised an outdoor breeding herd, composed of approximately 800 farrowing sows, plus five indoor rearing sites supplied by this herd. Weaners and grower age pigs in all of these rearing barns were sampled in the study. In each barn, these two juvenile age groups were accommodated in up to six straw-bedded pens, each containing 190 to 300 animals. All group medication had been discontinued at least two years before the first visit, leaving only individual treatments in cases of clinical disease. It had low AM usage, amounting to 10.8 mg of AM drugs per population correction unit (PCU) in 2017, compared with a contemporaneous average for the UK pig industry of 131 mg/PCU [24].

Farm 2 (F2) was solely an indoor rearing unit, receiving weaned pigs at 28 days of age from several breeding herds, including F1. There were up to 12 groups of 180 to 200 pigs in straw-bedded pens, with a new group placed on the farm every week, and they remained there until 10 weeks of age (around 30 kg) when they were transferred to a finisher unit. F2 had substantially higher AM usage in 2017 (170 mg/PCU) than F1 because pigs selected for F2 had delayed growth or evidence of clinical disease (such as diarrhoea) and often required AM treatment under veterinary advice. The two farms were approximately 20 km apart and did not share personnel or equipment.

On F2, AMs were administered as group treatments in feed as well as individually (by injection) where necessary. The drug(s) varied according to age and time point; they included apramycin, ampicillin, florfenicol, and sulphamethoxazole-trimethoprim. Between the final two sampling visits, a group of pigs was treated by injection with marbofloxacin after an outbreak of severe enteric disease with elevated mortality.

### 2.2. Faeces Sampling

An initial exploratory visit (Ex) was made to F1 in November 2017, collecting faecal samples from gilts, farrowing sows, and dry sows at the breeding site and from young stock (weaners and grower pigs) on rearing sites. Thereafter, four visits (T1 to T4) were made between March 2018 and October 2019 to both F1 and F2. Not every rearing unit of F1 was sampled on every occasion. At each visited site all pens occupied at the time by weaners or growers were sampled on these four occasions, with representative samples also taken from the breeding herd from F1. Because of stock turnover, it was not possible to re-sample the same groups repeatedly. Each sample was a pool of ten individual 1 g portions of formed stool collected from the floor of an individual pen, so each pool represented one occupied pen.

### 2.3. Laboratory Testing

#### 2.3.1. Isolation and Counts

Pools (10 g) of 10 samples were initially diluted 10-fold in 0.1 M phosphate-buffered saline (PBS, pH 7.2), and three subsequent serial decimal dilutions in PBS were performed. An aliquot (100 µL) of each dilution was spread onto ChromAgar ECC plates (CHROMagar™, Paris, France) to estimate the concentration (CFU/g) of *E. coli* in each pool [25]. Additionally, 100 µL of each dilution was spread onto two ChromAgar ECC plates supplemented with either 1 mg/L of ciprofloxacin (CIP) or 1 mg/L of cefotaxime (CTX). These are AMs from two classes employed therapeutically in pig medicine but also deemed HPCI in human medicine [8]. *E. coli* colonies were identified presumptively by their colour, and the concentrations of *E. coli* in the original sample were determined for each sample using the dilutions that yielded countable colonies on media with and without added AMs. The estimated proportions of *E. coli* resistant to CIP and CTX were calculated by dividing the concentrations derived from the respective AM-containing media by those derived from the corresponding non-AM-containing medium.

Representative presumptive *E. coli* colonies were sub-cultured to purity on their original isolation medium. Three colonies were picked from each dilution series on unsupplemented medium in order to capture a representative range and statistically robust number of phenotypic AMR variants. From each series on CIP or CTX plates, where a more focussed genomic analysis was planned, a single colony was picked per dilution series. All selected isolates were identified to species level by MALDI-TOFMS [26].

#### 2.3.2. Determination of Minimum Inhibitory Concentration (MIC)

MIC values were determined for a selection of the *E. coli* isolates originally grown on plain ChromAgar ECC. This was typically two isolates per pooled sample. These were purified by sub-culturing onto blood agar and incubating for 18 to 24 h. Thermo Fisher Trek^®^ (Thermo Fisher Scientific, Waltham, MA, USA) products were used to perform the MIC tests, using a broth microdilution method against a panel of 14 AMs employed in EU harmonised monitoring: ampicillin, azithromycin, cefotaxime, ceftazidime, chloramphenicol, ciprofloxacin, colistin, gentamicin, meropenem, nalidixic acid, sulfamethoxazole, tetracycline, tigecycline, and trimethoprim. *E. coli* ATCC 25922 was used for quality control. All MIC values were interpreted using epidemiological cut-off values (ECOFFs, Supplementary Table S1) issued by EUCAST [27]; i.e., AMR was defined as ‘non-wild type’ microbiological resistance. Designation of multi-drug resistance (MDR) used the European Food Safety Agency (EFSA) definition of resistance to three or more classes of AM [28].

### 2.4. Whole-Genome Sequencing (WGS)

WGS with genomic analysis was performed only on isolates selected from CIP plates, from pigs of all ages present on each farm. These represented strains likely to be circulating within each farm and resistant to a HPCI AM. These CIP-resistant strains were recovered consistently from all visits to both farms, a feature which facilitated between-farm and longitudinal molecular genetic comparisons. By comparison, CTX isolates were obtained only intermittently and so were not analysed in this manner. Genomic DNA was sequenced using Illumina HiSeq 4000 (Illumina Inc., San Diego, CA, USA), as previously described [29]. Species were confirmed using Kraken v1.0 and the MiniKraken DB_8 GB database [30]. Unicycler was used for *de novo* assembly [31] and *E. coli* seven-gene (Achtman) multi-locus sequence types were identified using the SRST2 tool [32]. The APHA SeqFinder pipeline was used to predict AMR phenotypes with approximately 98% accuracy [33]. It was considered that an AMR gene was present if there was 100% mapping of the raw sequence reads to the reference gene in the APHA SeqFinder database of AMR genes, allowing for up to 10 non-synonymous single-nucleotide substitutions.

‘Snippy’ version 4.3.6 [34] was used for the detection of single-nucleotide polymorphisms (SNPs), with *E. coli* K12 MG1655 (GenBank accession number U00096.3) used as the reference strain. A full pairwise SNP distance matrix was created using snp-dists v 0.6 [35]. Clones were identified as isolates exhibiting up to 14 SNP differences using the SNP distance matrix, as previously described [36]. A full-genome SNP alignment was generated and used to produce a 100-bootstrap maximum-likelihood phylogenetic tree with RAxML-NG [37], with *E. coli* K12 as the reference strain. The tree was rooted from the reference strain, and iTOL v5 [38] was used to render and annotate the tree. A dendrogram to visually investigate diversity among AMR profiles was generated using the packages ‘pheatmap’ and ‘viridisLite’ in RStudio v2021.09 [39].

### 2.5. Statistical Analysis

AMR characteristics of isolates from unsupplemented ChromAgar ECC plates of indoor rearing units of F1 and F2 at visits T1 to T4 were examined and compared. Confidence intervals for proportions of antimicrobial-resistant and multi-drug-resistant *E. coli* from sampled populations were calculated using the binomial distribution where the proportion was >0.1, and the Poisson distribution otherwise. Chi-square tests (or Fisher’s exact tests when the expected 2 × 2 contingency table values were <10) were used to investigate whether the percentages of antimicrobial-resistant and multi-drug-resistant *E. coli* isolates from young stock differed according to origin, i.e., F1 versus F2. A chi-square test for trends was used to examine changes in multi-drug-resistant isolates over time.

For each of the two AMs incorporated into isolation media (CIP or CTX), box plots for the counts and proportions of resistant bacteria CFU were done using ‘R’ (R Software, version 3.0.2). The Kruskal–Wallis test was used to compare the proportions of resistant *E. coli* between farms, from all visits, for CIP and (separately) for CTX. Statistical analyses were performed using STATA 15, applying the threshold of *p* < 0.05 for statistical significance.

## 3. Results

### 3.1. Sampling

The number of pens that could be sampled for fresh faeces at any given visit was dependent upon variations in occupancy. The modal number of sample pools per epidemiological group (dry sows, farrowing sows, gilts, growers, and weaners) per visit was six, with a range of zero to nine. Table 1 summarises the details on farm visits and samples. Over the five visits to F1, 1360 individual faecal samples were collected, resulting in 136 pools of 10 subsamples. From the four visits to F2 (T1 to T4), 440 individual faecal samples were collected, resulting in 44 pools.

### 3.2. Number and Proportion of E. coli Resistant to Ciprofloxacin and Cefotaxime

*E. coli* growth was observed on unsupplemented ChromAgar ECC for all samples. On CIP-supplemented agar, 95 pools (70%) from F1 and 29 pools (66%) from F2 yielded colonies. By contrast, on CTX plates, growth was seen from just 20 pools (15%) from F1 and from five pools (11%) from F2. For pools showing growth on AM-supplemented plates from either farm, the proportion of CIP- or CTX-resistant *E. coli* against reference counts from plain plates was consistently low (Supplementary Figures S1 and S2) and not significantly different between the farms when visit data were combined (Kruskal–Wallis test, *p* = 0.139 and *p* = 0.779 for CIP and CTX, respectively).

### 3.3. Antimicrobial Resistance Panel

From the sampled F1 rearing sites, there were 12, 24, 28, and 18 isolates from plain ChromAgar ECC tested from visits T1 to T4, respectively. Forty eight of these 82 isolates, or 59% (95% CI: 47–69%), showed resistance to at least one AM.

**Figure 1:**
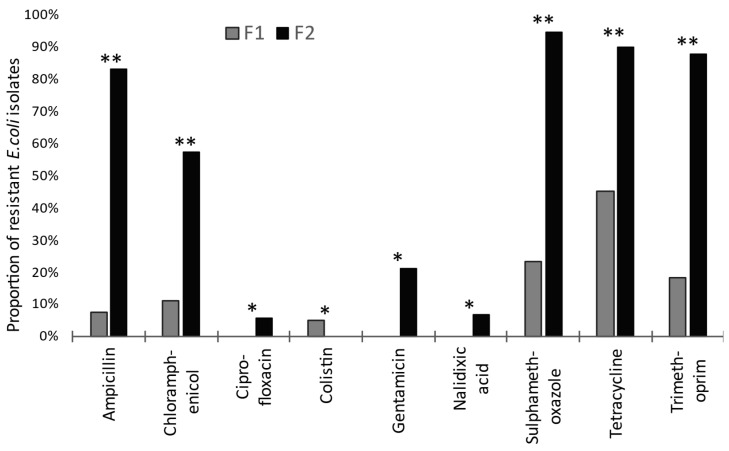
Proportions of microbiologically resistant isolates for the nine (of 14) antimicrobials showing a significant difference between rearing herds on ‘low’ (F1) versus ‘high’ (F2) antimicrobial-drug-usage farms. Significant differences are indicated: * *p* < 0.05 and ** *p* < 0.001. (For azithromycin, cefotaxime, ceftazidime, meropenem, and tigecycline, differences were not significant.)

From F2, 105 *E. coli* were tested from T1 to T4, comprising 24, 24, 33, and 24 isolates, respectively. Resistance to at least one of the 14 AMs was seen in 101 of these, or 96% (95% CI: 91–99%). Comparing young stock on both premises, the proportion of resistant *E. coli* from F1 was significantly lower (*p* < 0.0001) than from F2. Significant differences for resistance to specific AM drugs are shown in Figure 1. Colistin was the only AM where more frequent resistance was seen on F1 than on F2 (Figure 1), albeit such resistance was exhibited by only seven isolates. These were obtained from F1 on one occasion (T3) from three pooled samples, each representing a different production stage: weaners (one isolate), growers (three isolates), and farrowing unit (three isolates).

**Figure 2:**
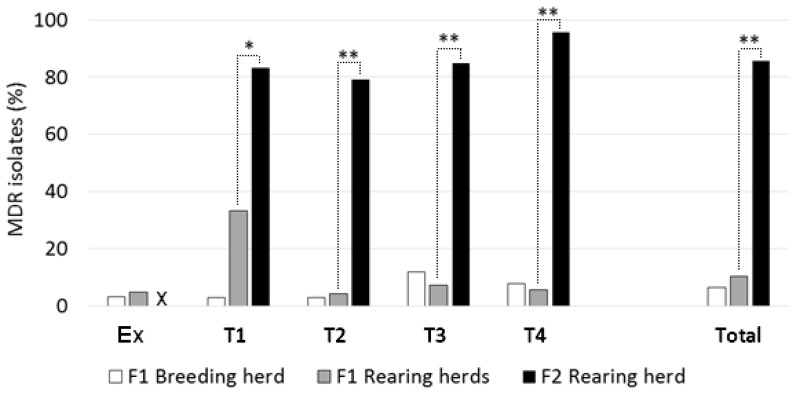
Proportions of multi-drug-resistant *E. coli* from ‘low’ (F1) versus ‘high’ (F2) antimicrobial-drug-usage farms, at individual sampling visits (Ex to T4) and in total. Dotted lines show comparisons made between rearing herds, with significant differences indicated: * *p* < 0.05; ** *p* < 0.001. ‘X’: no Ex visit to F2.

MDR was seen in 8 of 82 or 9.8% (95% CI: 4.2% to 19%) of young stock isolates from F1 samples. From F2, 90 isolates or 86% (95% CI: 78% to 92%) showed MDR, which was a markedly higher proportion (*p* < 0.0001). Further details, by visit number, are shown in Figure 2. For F1, there was no significant longitudinal trend in the proportion of multi-resistant isolates per visit from Ex to T4.

### 3.4. Whole-Genome Sequencing

**Figure 3:**
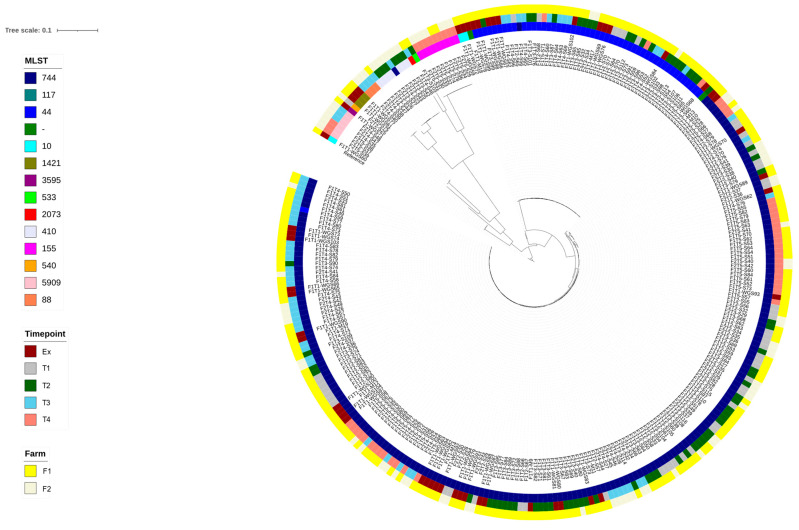
Phylogenetic tree of *E. coli* isolates from farms 1 (low antimicrobial use) and 2 (high antimicrobial use), constructed following full-genome single-nucleotide polymorphism alignment, using *E. coli* K12 as a reference. ‘MLST’ = multi-locus sequence type. For time point details, see Table 1.

WGS resulted in 214 reads from F1 and 81 from F2. These have been deposited in the NCBI archive (BioProject PRJNA1033605). The SNP-based phylogenetic tree (Figure 3) showed isolates clustering almost entirely according to their multi-locus sequence type (ST). Twelve STs were identified, plus an outlier group of three ST117 isolates that had over 60,000 SNP differences to all other isolates. These three were the only isolates of ST117, and so the tree was redrawn to exclude this cluster. There was no clear clustering of isolates based on farm or time point.

Three STs (44, 155, and 744) were recovered from both F1 and F2, with the dominant STs being 744 and 44 (69.5% and 17.6 of sequenced isolates, respectively). There was some genetic heterogeneity within each of these STs, as shown by the SNP distance matrix: ST44 isolates (n = 52) had a range of 8 to 354 SNPs (mean of 86), whereas ST744 isolates (n = 205) were more diverse with a range of 2 to 15,254 SNPs and a mean of 891. However, there were several sub-clades within the ST744 group that were more genetically homogeneous. Based on clones showing up to 14 SNP differences, some clonal groups within ST744 were present on both farms, and clones were also isolated on multiple sampling occasions.

The dendrogram based on AMR determinants (Supplementary Figure S3) allowed a comparison of the diversity of AMR profiles between F1 and F2. As with the SNP phylogenetic tree, isolates mostly clustered according to their ST. However, whilst there were 131 different resistance profiles, only two of these were present in sequences from both F1 and F2. Furthermore, isolates from F2 showed a greater diversity of AMR profiles: among 210 isolates from F1, there were 55 unique resistance profiles and an average of 7.8 AMR genes, whereas among 80 F2 isolates, there were 74 unique resistance profiles, with an average of 8.6 AMR genes.

Isolates of the most common ST (744) formed three distinct clusters. A previous study [36] had identified a transposon encoding MDR that also harboured heavy metal and biocide resistance genes and was integrated in the chromosome of ST744 isolates from F1 at time points T1–T3. Further examination of the gene content based on the clustering of ST744 isolates in the dendrogram indicated that this genomic island was present on both farms and at multiple time points.

The second most common ST (44) aggregated two distinct clusters on the dendrogram. Cluster 1 isolates were isolated from both F1 and F2, exhibited chromosomal fluoroquinolone (ciprofloxacin) resistance mutations, and harboured an AMR gene cassette encoding resistance to aminoglycosides, sulphonamides, and trimethoprim. By contrast, members of Cluster 2 were only isolated from F1 and showed chromosomal fluoroquinolone resistance mutations but did not carry the multiple-resistance gene cassette. Both groups included isolates from all five time points.

## 4. Discussion

The present study exploited an opportunity to examine AMR among indicator bacteria (*E. coli*) from a pig farm that had a strategy of minimising AM usage, and also from another farm that was linked to the first by stock transfers. The two farms were distinguished by markedly different AM usage with respect to frequency, administration routes, and agents employed. AMR at the population level was the focus; therefore, the sampling strategy treated pens of animals as the unit for investigation. Temporal patterns on farms were examined by repeated sampling over two years, and relationships between circulating strains were analysed using WGS.

Using one pool of 10 voided faeces subsamples per pen for culture permitted a resource-efficient periodic broad survey of the *E. coli* strains present within large groups of young stock on each unit. This approach did however preclude certain more detailed and sensitive analyses, for example, examining strain persistence or variation in individuals over time or after AM administration. This would have required more frequent individual or small-pool faeces samples from smaller segregated groups. Although the availability of occupied pens varied between visits (as is often unavoidable with field surveys), similar numbers of sampled pens were represented from each farm in aggregated data from all visits for statistical comparisons.

AM drug use in pig production in the UK has declined substantially in recent years, for example, from 278 to 110 mg/PCU between 2015 and 2019 [40], although it is currently unclear whether the prevalence of resistance on farms has altered in consequence. We can compare the AMR prevalence values from the present phenotypic data with contemporaneous (2017) UK-wide survey data for fattening pigs at slaughter, obtained under EU harmonised monitoring and using the same 14-AM screening panel plus ECOFF interpretation breakpoints as in the present work ([41], p. 77). Although the study populations and sampling methodology in the current work and the UK-wide survey are somewhat different, it is noteworthy that fattening stock on F1 (which had lower AM usage than the UK average) yielded a higher proportion of fully susceptible *E. coli* (49%) than was seen in the UK-wide data (32%). Similarly, the proportion of F1 fattener isolates showing MDR (8.7%) was substantially lower than the values of 35% *E. coli* in aggregated EU data for fattening pigs in 2017 [41], or 36% in the UK, 2017 to 2019 data for fattening pigs, poultry, and calves using ECOFF breakpoints [40].

It is plausible that these differences to some extent arise from lower annual AM usage on F1 (10.8 mg/PCU) than in the UK pig industry overall (131 mg/PCU) [40], with AMs also only being administered on F1 by injection of individual animals. Treating individuals, compared with group medication, was associated with lower frequencies of resistance for some AMs in a study by Dunlop et al. [11]. By contrast, for the higher-AM-use farm (F2), the corresponding proportions of fully susceptible (4%) and multi-drug-resistant (86%) *E. coli* were substantially lower and higher, respectively, than the UK and EU survey values discussed above. As the animals on F2 were more likely to require AM treatment, and group administration of AMs was employed, it seems likely that these higher frequencies of resistance are related to the increased selective pressure exercised by AM use [42].

However, whilst AM exposure clearly has a potential causal relationship with the observed differences, it is also probable that there are several confounding factors that may impact the degree of AMR seen in sentinel *E. coli*. Whilst both farms were part of the same production pyramid, pigs on F2 were also sourced from other premises. These other sources were nonetheless within the same pyramid as F1 and F2 and had lower AM usage than F2. The selection of animals in poor health for F2, and the further stresses of their transfer and mixing, may also be factors in the observed correlations between farm and AMR, impacting the characteristics of individual enteric microbiomes and potentially enhancing horizontal AM gene transfers [43]. There is an example of such a phenomenon where pigs given apramycin and then exposed to environmental stress showed more prolonged shedding of *E. coli* with elevated resistance to apramycin than their unstressed counterparts did [44].

In addition, compared to a multi-premises survey, the present two-farm study is more vulnerable to biases that arise from individual differences in management on the two farms, from specific features of the farms’ relationships to each other, and from differences between these farms and UK pig-rearing units more broadly. The overall strength and direction of these biassing factors is unknown, but caution should be exercised, particularly when contemplating the generalisability of the findings to farms with differing management systems.

Nonetheless, the present observations on AMR are consistent both with our hypothesis that AM usage correlates with frequencies of individual resistances (and with MDR) and with findings reported by Österberg et al. [45] concerning indicator *E. coli* from market-age pigs in Sweden, France, Denmark, and Italy. They reported that, within their study population, presumed lower-AM-usage farms (organic and/or Swedish) showed significantly less frequent phenotypic resistance to several commonly used AMs. A similar lower frequency of AMR in low (zero) AM usage versus conventional pig production has been reported in recent years from North America, among both *E. coli* [46] and *Salmonella* spp. [47].

When considering the specific AM resistances that were found to be significantly different between F1 and F2, any causal inferences must be tentative, given that we were not sampling specific animals or groups with known exposure to particular AMs. Nonetheless, many resistances are seen to correlate with expected differences in selective pressure on the units as a whole, based upon recent recorded uses of specific drugs or drug classes. This is the case for higher frequencies of resistance on F2 to ampicillin, sulphamethoxazole, trimethoprim, nalidixic acid, and ciprofloxacin. Furthermore, cross-resistance is recognised between florfenicol (used on F2) and chloramphenicol (significantly more common resistance on F2), associated typically with *floR* [48]. This gene, encoding an efflux pump, was identified amongst the present *E. coli* isolates (Supplementary Figure S3). Chloramphenicol-specific resistance genes *cml*, *cmlA1*, and *catA1* were also found, which may show co-selection with other resistance genes under AM pressure.

Similarly, elevated gentamicin resistance on F2 is consistent with the use of apramycin, which would be expected to select for the plasmid-borne *aacC4*, encoding AAC(3)-IVa acetyl transferase. This also confers gentamicin resistance [49,50] and was present among the sequenced isolates (Supplementary Figure S3). AMs not used on F2, either recently or at all, did not show a difference in frequency between the farms, except for tetracycline. The tetracycline class is commonly used in pig production, and *tet* resistance genes were often found in the whole-genome sequences. Where *tet* genes were present in the same genome as resistance determinants selected by on-farm AM use, then co-selection would occur, resulting in elevated frequencies of tetracycline resistance.

Co-selection of resistance is a characteristic of MDR, and there was a marked difference in the proportion of multi-drug-resistant *E. coli* among young stock between F1 (8.7%) and F2 (86%). Experimentally, higher pig *E. coli* MDR risk has been reported in association with in-feed AMs, with the largest effect being observed during the nursery phase [51]. A survey of growing pigs on Canadian farms also indicated that in-feed AM treatment was a risk factor for *E. coli* resistance to three or more AMs, although the use of some AMs by injection was protective [13]. Furthermore, withdrawal of in-feed AMs (apramycin and trimethoprim) was followed by a decline in multi-resistant *E. coli* in a longitudinal study on a commercial pig unit [52]. The magnitude of the observed difference in MDR between farms in the present study is noteworthy, although, as discussed earlier for AMR, the influence of non-AM factors remains unclear. Enhanced AMR horizontal gene transfer, which can be influenced by several factors (including those not associated with administered AMs), could be a substantial contributor to the creation of multi-resistant enteric bacteria. Testing the prevalence values of MDR on F1 did not identify significant evidence of a temporal trend in this low-usage environment. However, given the number of samples per visit, the statistical power of this analysis was limited.

The isolation of CIP- and CTX-resistant bacteria on pig farms is not uncommon when using selective culture [53,54]. This is reflected in the present data by isolation from both farms of *E. coli* resistant to CIP and CTX from a majority (69%) and a substantial minority (14%), respectively, of sample pools. However, the proportion of isolates resistant to these AMs, expressed as a percentage of the *E. coli* population in pooled faeces, was very low throughout the study. A difference could not be detected between the two farms in this proportion of resistant colonies, nor did the proportion of resistant *E. coli* decrease significantly over two years. Given that there was minimal or zero recent use of either class of AMs on the studied units, amounting to one episode of fluoroquinolone use on F2 between T3 and T4, this low proportion of resistant *E. coli* and lack of difference in this respect between farms is unsurprising.

It has been reported that, for several AMs commonly used in pig production, *E. coli* resistances have persisted at diminished prevalence values on pig farms with low or zero AM pressure [45,46]. This can be linked to phenomena such as co-selection (by other drugs or heavy metals in animal feed), compensatory evolution, and the dissemination of resistance determinants on mobile genetic elements [2,21,23]. One AM class for which persisting resistance has been observed, both in the present study and elsewhere, is the extended-spectrum cephalosporins [21]. In the report by Abraham et al. [21], a plasmid-borne extended-spectrum beta-lactamase was identified, which may have assisted AMR persistence via horizontal transfer and co-selection mechanisms. Regardless of cause, the observed reservoirs of resistance to CIP and CTX on the current farms form a base from which the proportion of resistant *E. coli* could rise rapidly if either class of AM were to be used.

Despite the observed differences in the prevalence of AMR between F1 and F2, WGS of CIP-resistant isolates revealed some closely related multi-drug-resistant strains and also some clonal AMR populations of multi-locus ST744 that were recovered from both farms and which were also isolated from the same farm on more than one occasion. These results build on the findings from an earlier report of F1 [36], where clones of the dominant STs (44 and 744) were seen to persist on the premises for one year (that being the first year of the present study). The current work demonstrates the continued retrieval of these AM-resistant strains (typically harbouring several AMR genes) beyond that first year of investigation and shows that they were also present on F2. The persistence of these clonal *E. coli* on F1 was despite stock only moving from F1 to F2, i.e., from the lower- to the higher-AM-usage farm, which suggests that AMR was stably persisting on F1, potentially with some cycling between the outdoor breeding pigs and wild birds [36]. Another possibility, that AMR strains maintained by AM selection on F2 were back-transferred to F1 (via personnel, transporters, or other fomites), seems less likely given the biosecurity measures routinely employed in the pig industry. Samples from the environment of both farms, including wildlife, equipment, and transporters, would be needed to identify and clarify reservoirs of AMR and transmission routes.

Despite the presence of highly similar antimicrobial-resistant *E. coli* strains observed (using WGS) on the two farms, there were some differences detected between the AMR gene profiles of isolates from F1 versus F2, with the F2 strains showing more diversity. This cannot confidently be attributed to more varied AM pressures on *E. coli* in F2, although that may be a factor. Other causes, not least differences between F1 and F2 in the sources of incoming animals and the stability of their populations, are likely to have had an influence.

There is consistent evidence from the present report and from other discussed studies that reduced AM usage in pig production is associated with a reduced prevalence of AMR and MDR among *E. coli*. We may further assume tentatively that this holds for other enteric bacteria, given that there is a shared gut microbiome and similar exposure to AMs for all enteric microorganisms. Therefore, it is important to recognise that the reduction in, and avoidance of, AM usage in pig production (via high standards of husbandry, rational prescribing, and strictly limiting the use of critically important antimicrobials) are valuable tools to protect the efficacy of AMs in both veterinary and human medicine. However, we also observe that resistant bacterial strains, or at least resistance genes, tend to persist for months to years on production premises even in the absence of specific selection pressures. Potential mechanisms for this are recognised, including co-selection, adaptive fitness compensation, horizontal gene transfer, and strain transfer between co-managed premises. Therefore, we should also consider additional factors and measures that may counteract AMR. These include avoiding transfers of stock and equipment between premises, eliminating (as much as possible) interfaces between stock and wildlife, including birds, and considering the possibility that co-selection may occur because of poorly effective disinfection.

In summary, a detailed longitudinal study of faecal pools from two pig farms linked by unidirectional movement of young stock showed that a fifteen-fold difference in the population-corrected AM usage was positively correlated with a significant difference in the proportion of antimicrobial-resistant *E. coli*. Furthermore, multi-resistant *E. coli* were markedly more prevalent on the higher-usage farm than on the lower-usage farm. A novel observation, arising from the detailed and longitudinal focus on two farms, was that some genetically similar multi-drug-resistant strains plus some clonal or highly similar antimicrobial-resistant isolates were found to be present on both of the study farms regardless of AM usage, and such strains were recovered from the low-usage farm throughout the two years of the study. It appears that, whilst there may be a lower prevalence of AMR on premises with reduced AM usage, there can be a persistent reservoir of antimicrobial-resistant strains with the potential for selection and amplification. In the present study, these included strains resistant to critically important AM drugs.

## Figures and Tables

**Table 1:** Number of pooled faecal samples collected from different age groups at each time point.

Sampling Occasion	F1 Breeding Site			F1 Rearing Sites		F2 Rearing Site		Total
	G	DS	FS	W	G	W	G	
Ex *: November 2017	6	6	6	6	5	0	0	29
T1: March 2018	6	6	6	0	6	7	5	36
T2: October 2018	6	6	6	6	6	6	6	42
T3: March 2019	6	6	5	6	6	9	3	41
T4: October 2019	6	6	6	6	0	8	0	32
Total	30	30	29	24	23	30	14	180

F1 and F2 were ‘low’ and ‘high’ antimicrobial-use farms, respectively. G = gilts, DS = dry sows, FS = farrowing sows, W = weaners, GF = growers/finishers. * initial exploratory visit.

## Data Availability

The original data presented in the study are openly available in NCBI archive at https://www.ncbi.nlm.nih.gov/bioproject/?term=PRJNA1033605 (accessed on 18 March 2026) reference number BioProject PRJNA1033605.

## Supplementary Materials

The following supporting information can be downloaded at: https://www.mdpi.com/article/10.3390/vetsci13040309/s1, Figure S1: Box plots showing the distribution of *E. coli* in all age groups across different sampling visits to Farm 1; Figure S2: Box plots showing the distribution of *E. coli* in all age groups across different sampling visits to Farm 2; Figure S3: Dendrogram based on AMR determinants in *E. coli* isolates from two pig farms, F1 and F2, with low and high antimicrobial drug usage, respectively; Table S1: Epidemiological cut-off values for *E. coli* used in AMR determination.

## Abbreviations

The following abbreviations are used in this manuscript:

AM	Antimicrobial
AMR	Antimicrobial resistance
CFU	Colony-forming units
CIP	Ciprofloxacin
CTX	Cefotaxime
ECOFF	Epidemiological cut-off value
EU	European Union
F1	Farm 1 (lower antimicrobial usage)
F2	Farm 2 (higher antimicrobial usage)
HPCI	Highest priority critically important
MALDI-TOFMS	Matrix-assisted laser desorption/ionisation–time of flight mass spectrometry
MDR	Multi-drug resistance
MIC	Minimum inhibitory concentration
PBS	Phosphate-buffered saline
PCU	Population Correction Unit
SNP	Single-nucleotide polymorphism
ST	Sequence type (multi-locus)
WGS	Whole-genome sequencing
