# Supplementary material for: Closely Related Escherichia coli Strains with Multiple Resistances Found on Co-Managed Pig Farms Despite Marked Differences in Farm Antimicrobial Drug Usage
Source: Vet Sci. 2026 Mar 24;13(4):309. doi: 10.3390/vetsci13040309 (PMC13120469; doi:10.3390/vetsci13040309)
Supplement: Supplementary file 1 [file vetsci-13-00309-s001.zip › Pig AMR Suppl fig S1.pdf]

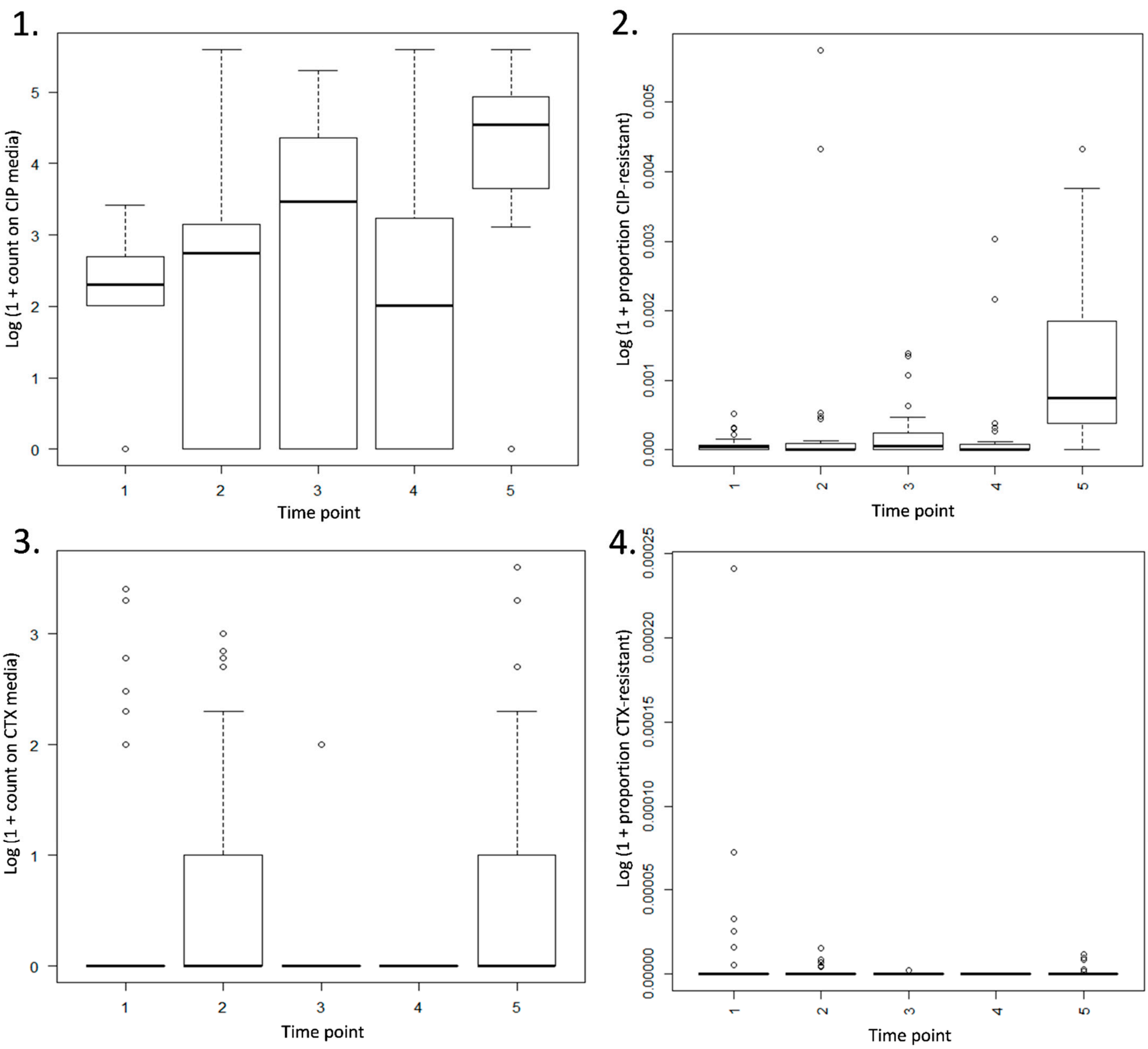

Supplementary Figure S1: Box plots showing the distribution of *E. coli* in all age groups across different sampling visits to Farm 1.

1 and 3: *E. coli* counts (CFU/g) from ciprofloxacin (CIP) and cefotaxime (CTX) media.

2 and 4: proportions of resistant *E. coli* from CIP and CTX media.

Medium was ChromAgar ECC, plain or supplemented with 1 mg/L of either CIP or CTX.
