# Supplementary material for: Closely Related Escherichia coli Strains with Multiple Resistances Found on Co-Managed Pig Farms Despite Marked Differences in Farm Antimicrobial Drug Usage
Source: Vet Sci. 2026 Mar 24;13(4):309. doi: 10.3390/vetsci13040309 (PMC13120469; doi:10.3390/vetsci13040309)
Supplement: Supplementary file 1 [file vetsci-13-00309-s001.zip › Supplementary Table S1.pdf]

Table S1: Epidemiological cut-off values for *E. coli* used in AMR determination.

**Antimicrobial**	**ECOFF** (mg/L)
Ampicillin	> 8
Azithromycin	> 16
Cefotaxime	> 0.25
Ceftazidime	> 0.5
Chloramphenicol	> 16
Ciprofloxacin	> 0.064
Colistin	> 2
Gentamicin	> 2
Meropenem	> 0.125
Nalidixic acid	> 16
Sulphamethoxazole	> 64
Tetracycline	> 8
Tigecycline	> 1
Trimethoprim	> 2
