# Supplementary figures and images for: Closely Related Escherichia coli Strains with Multiple Resistances Found on Co-Managed Pig Farms Despite Marked Differences in Farm Antimicrobial Drug Usage
Source: Vet Sci. 2026 Mar 24;13(4):309. doi: 10.3390/vetsci13040309 (PMC13120469; doi:10.3390/vetsci13040309)

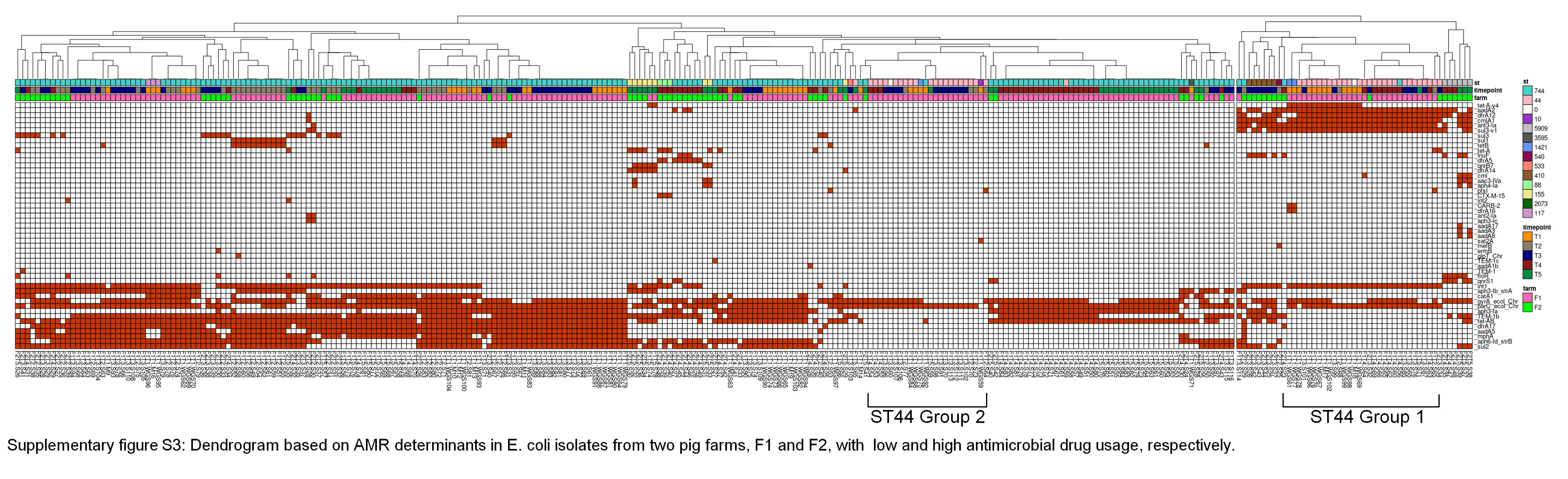

Supplement: Supplementary file 1 [file vetsci-13-00309-s001.zip › Pig AMR Suppl fig S3.jpg]
